# Hair Growth Stimulation Effect of *Centipeda minima* Extract: Identification of Active Compounds and Anagen-Activating Signaling Pathways

**DOI:** 10.3390/biom11070976

**Published:** 2021-07-02

**Authors:** Byoung Ha Kim, Myong Jin Lee, Won-Yung Lee, Jaesung Pyo, Myoung-Sook Shin, Gwi Seo Hwang, Dongchul Shin, Chang Eop Kim, Eun-Seok Park, Ki Sung Kang

**Affiliations:** 1 D. Nature Co., Ltd., Seongnam 13174, Korea; mot37@d-nature.co.kr; 2 College of Korean Medicine, Gachon University, Seongnam 13120, Korea; myongene@naver.com (M.J.L.); wonyung21@naver.com (W.-Y.L.); ms.shin@gachon.ac.kr (M.-S.S.); seoul@gachon.ac.kr (G.S.H.); sdc2510@gmail.com (D.S.); eopchang@gachon.ac.kr (C.E.K.); 3 College of Pharmacy, Kyungsung University, Busan 48434, Korea; jspyo@ks.ac.kr; 4 School of Pharmacy, Sungkyunkwan University, Suwon 16419, Korea

**Keywords:** *Centipeda minima*, human hair follicle dermal papilla cells, Wnt/β-catenin signaling, VEGF

## Abstract

*Centipeda minima* (L.) A. Braun & Asch is a well-studied plant in Chinese medicine that is used for the treatment of several diseases. A recent study has revealed the effects of extract of *Cetipeda minima* (CMX) standardized by brevilin A in inducing hair growth. However, the mechanism of action of CMX in human hair follicle dermal papilla cells (HFDPCs) has not yet been identified. We aimed to investigate the molecular basis underlying the effect of CMX on hair growth in HFDPCs. CMX induced the proliferation of HFDPCs, and the transcript-level expression of Wnt family member 5a (Wnt5a), frizzled receptor (FZDR), and vascular endothelial growth factor (VEGF) was upregulated. These results correlated with an increase in the expression of growth-related factors, such as VEGF and IGF-1. Immunoblotting and immunocytochemistry further revealed that the phosphorylation of ERK and JNK was enhanced by CMX in HFDPCs, and β-catenin accumulated significantly in a dose-dependent manner. Therefore, CMX substantially induced the expression of Wnt signaling-related proteins, such as GSK phosphorylation and β-catenin. This study supports the hypothesis that CMX promotes hair growth and secretion of growth factors via the Wnt/β-catenin, ERK, and JNK signaling pathways. In addition, computational predictions of drug-likeness, together with ADME property predictions, revealed the satisfactory bioavailability score of CMX compounds, exhibiting high gastrointestinal absorption. We suggest that CMX could be used as a promising treatment for hair regeneration and minimization of hair loss.

## 1. Introduction

Hair loss or alopecia is an incurable disease that affects the quality of life and is emotionally distressing, especially regarding self-confidence. Hair loss is caused by physiological situations related to hormonal imbalance, age, autoimmune conditions, medications, and genetics [1]. Androgenetic alopecia (AGA) is a pattern of hair loss that affects both men and women [2]. AGA arises as the result of stepwise miniaturization of the hair follicle and alteration of hair-cycle dynamics [3].

Hair growth and hair loss in mammalian species are also controlled by a follicular cell cycle that includes periods of growth (anagen phase), regression (catagen phase), and rest (telogen phase) [4]. These cyclic changes involve rapid remodeling of the epithelial and dermal components of hair follicles. The hair growth cycle requires reciprocal interactions between mesenchymal and epithelial cells in the hair follicles [5]. Hair follicles consist of various lineages of epithelial cells surrounding the hair shaft, with mesenchymal cells aggregated in the dermal papilla. During hair growth cycling, complicated crosstalk/interactions occur between epithelial and mesenchymal cells, which regulate the proliferation and differentiation of epithelial cells [6,7].

*Centipeda minima* (L.) A. Braun & Asch. is plant in the Asteraceae family widely distributed in China, Korea, Japan, Australia, and India, and is commonly used Chinese herbal medicine. Aerial parts of *C. minima* have been used for centuries to treat diarrhea, asthma, nasal allergies, and malaria. Recent pharmacological studies on an extract of *C. minima* have demonstrated that it has antioxidant, neuroprotective, antibacterial, and anticancer biological activities. Twelve major chemical constituents such as flavonoids, polyphenolic acid, and sesquiterpene lactones have been identified through HPLC-Q-TOF-MS [8,9]. Previously, we evaluated the effects and mechanisms of action of a standardized emulsion extract of *C. minima* (CMX) by brevilin A on hair loss using a framework that integrated an in vitro investigation, a clinical study, and a network pharmacology-based analysis [10,11]. The clinical study showed that total, terminal, and anagen hair counts were significantly higher in the CMX group than in the placebo group, suggesting that CMX is an effective treatment. Moreover, the network pharmacology-based approach identified the gene targets of CMX and their potential mechanisms, focusing on the JAK-STAT signaling pathway. This study suggested that the medicinal herbal mixture CMX could be useful in the treatment of mild to moderate vertex balding and resulted in favorable effects on hair quality that contributed to visible improvements in hair growth in treated patients. In this study, we investigated the mechanism by which CMX affects hair growth via the Wnt/β-catenin signaling pathway.

Based on the connections between Wnt/β-catenin activation and hair development, researchers have identified the activators of this signaling pathway as potential interventions for hair therapy. Many natural and synthetic compounds have been reported to promote hair regeneration through the activation of Wnt/β-catenin signaling [12]. However, the number of people suffering from hair loss is increasing despite the development of therapies. Therefore, it is crucial to develop new treatment strategies to combat hair loss and increase hair proliferation. Therefore, in this study, we investigated the mechanism of hair regeneration which activates the Wnt/β-catenin pathway, and the therapeutic effects of CMX, a medicinal herbal extract that stimulates hair regrowth.

## 2. Materials and Methods

### 2.1. Antibodies and Reagents

Antibodies against β-catenin (D10A8), GSK3β (27C10), phospho-GSK3β (Ser 9), p38(A-12), phospho-p38 Thr180/Tyr182 (D3F9), ERK1/2 (p44/42), phospho-ERK (Thr202/Tyr204), SAPK/JNK, phospho-JNK (Thr183/Tyr185), AKT (5G3), phospho-AKT (Ser473), and GAPDH were purchased from Cell Signaling Technology (Danvers, MA, USA). Additionally, antibodies against IGF (W18) and VEGF (C-1) were purchased from Santa Cruz Biotechnology (Santa Cruz, CA, USA). Goat anti-rabbit IgG cross-adsorbed secondary antibody, Texas Red-X, and SlowFade Gold antifade reagent with DAPI were obtained from Invitrogen (Carlsbad, CA, USA). Cell culture slides for immunofluorescence were purchased from SPL (Gyeonggi-do, Korea).

### 2.2. Plant Materials and Preparation of CMX

*C. minima* was purchased in December 2019 from Natural-herb (Goesan, Korea). The material was identified by 1 of the authors (J.P.). A voucher specimen of the material (CM-2019-001) was deposited in the herbarium at Kyungsung University. CMX was prepared by D. Nature Co., Ltd. (Seongnam, Korea) using the efficient separation of brevilin A from *C. minima* by inducing phase separation in the emulsion as reported previously [11], and its International Nomenclature Cosmetic Ingredient ID number is 33849.

### 2.3. High-Performance Liquid Chromatography (HPLC)

The amounts of major compounds of the CMX were determined by HPLC-UV (Thermo Scientific Dionex Ultimate 3000, Thermo Fisher Scientific, Sunnyvale, CA, USA). For the HPLC conditions, refer to the previous study [11]. Filtered samples (10 µL) were injected into a SUPERSIL column ODS-I (250 mm × 4.6 mm, 5.0 µm), and the column temperature was maintained at 40 °C. For the mobile phase, 0.1% formic acid in distilled water was used as Solvent A and methanol was used as Solvent B. The composition of the solvent was (A) 45% and (B) 55%, and an isocratic composition was used. The measured UV wavelength was 224 nm, and the flow rate of the mobile phase was 1 mL/min.

### 2.4. Cell Culture

Human hair follicle dermal papilla cells (HFDPCs) were purchased from PromoCell (Heidelberg, Germany). The cells were maintained in a follicle dermal papilla cell basal medium (PromoCell) supplemented with 4% fetal calf serum, 0.4% bovine pituitary extract, 1 ng/mL basic fibroblast growth factor, and 5 μg/mL recombinant human insulin (PromoCell) at 37 °C in a humidified atmosphere of 5% CO_2_.

### 2.5. Cell Viability Assay

Cell viability was determined using an EZ-Cytox assay kit (DoGenBio, Seoul, Korea) [13]. Briefly, HFDPCs were seeded into 96-well plates at a density of 2 × 10^4^ cells per well. After 24 h of incubation, the cells were treated with CMX at various concentrations of 0.39, 0.78, 1.56, 3.13, 6.25, and 12.5 μg/mL for 24 h. The cells were incubated with water-soluble tetrazolium salt (WST-1) for 1 h. The absorbance was measured at 450 nm using an enzyme-linked immunosorbent assay (ELISA) plate reader, and the results were expressed as percentages of the untreated controls.

### 2.6. RNA Isolation and Quantitative Real-Time Polymerase Chain Reaction (qRT-PCR)

Total RNA was isolated from cells using the RNeasy Mini Kit (Qiagen Inc., Valencia, CA, USA) and quantified using the Nanodrop method. cDNA was synthesized using the AccuPower RocketScript Cycle RT Premix (Bioneer, Daejeon, Korea), and 1 µg of cDNA was used for PCR.

qRT-PCR was performed using a PowerUP SYBR Green PCR Master Mix (Thermo Fisher Scientific) with a QuantStudio 3 Real-Time PCR System (Thermo Fisher Scientific) according to the manufacturer’s protocol [14]. The mRNA expression level of each gene was calculated using the 2^−ΔΔCt^ method and normalized to that of glyceraldehyde-3-phosphate dehydrogenase (GAPDH). The sequences of the primers used in qRT-PCR are listed in Table 1. The cycling conditions were as follows: 10 min at 95 °C for enzyme activation, denaturation for 15 s at 95 °C, and annealing for 60 s at 60 °C. qRT-PCR was performed for at least 35 cycles.

### 2.7. ELISA

The expression levels of KGF/FGF-7 (keratinocyte growth factor/fibroblast growth factor-7) and VEGF (vascular endothelial growth factor) in the culture medium were measured; 500 mL of the culture supernatant was harvested from the cultured cells. The levels of KGF/FGF-7 and VEGF were measured using an ELISA kit (R&D Systems, Minneapolis, MN, USA) according to the manufacturer’s instructions. Absorbance was determined at 450 nm using a microplate reader.

### 2.8. Preparation of Cell Lysates and Immunoblotting

HFDPCs were treated with the indicated concentrations of CMX for 24 h. Following treatment, the cells were washed with phosphate-buffered saline (PBS) twice and then lysed in a cold radioimmunoprecipitation assay (RIPA) buffer (Rockland, Limerick, PA, USA) containing 1 mM DTT (Sigma, Saint Louis, MO, USA), a phosphatase inhibitor cocktail (Merck, Darmstadt, Germany), and a protease inhibitor cocktail (Roche Diagnostics Corp., Indianapolis, IN, USA) [15]. The protein concentration of each sample was adjusted to a constant value after measurements using the BCA Protein Assay Kit (Thermo Scientific, Rockford, IL, USA). Next, cell lysates were resolved on an 8−10% sodium dodecyl sulfate-polyacrylamide gel and proteins were subsequently transferred to an Immobilon-P PVDF membrane (Millipore, Billerica, MA, USA). The membrane was blocked with 5% skim milk prepared in Tris-buffered saline (TBS, pH 7.4) containing 0.1% Tween 20 (TBS-T) for 120 min and probed with specific primary antibodies overnight at 4 °C. The membrane was then incubated with horseradish peroxidase-conjugated secondary antibody for 1 h and washed with TBS-T 3 times before visualization using an ECL system.

### 2.9. Immunofluorescence Staining

HFDPCs were seeded in a 4-well chamber (2 × 10^4^ cells/well) and cultured in a growth medium containing the supplement mixture. Cells were fixed in 4% paraformaldehyde diluted in PBS for 15 min at room temperature. After blocking with a blocking reagent containing 5% BSA and 0.1% Triton X-100 in PBS for 60 min at room temperature, the cells were stained with β-catenin antibody (1:100) (Cell Signaling Technology, Danvers, MA, USA) overnight. The cells were rinsed 3 times with PBS and incubated with Texas Red-X goat anti-rabbit antibody for 2 h at room temperature. Cells were counterstained with DAPI and observed using a TH4-200 immunofluorescence microscopy system (Olympus, Tokyo, Japan).

### 2.10. Assessment of Pharmacokinetic and Drug-Likeness Properties

The pharmacokinetic and drug-likeness properties of CMX were predicted using the web-based tool SwissADME (http://www.swissadme.ch/, accessed on 13 April 2021). The SwissADME computational tool allows the prediction of the following pharmacokinetic characteristics: gastrointestinal (GI) absorption, inhibition of some cytochrome P450 (CYP) enzymes regularly involved in interactions with xenobiotics (CYP1A2, CYP2C19, CYP2C9, CYP2D6, and CYP3A423), and skin permeability, with an accuracy of 71 to 89% [16]. The drug-likeliness profile of CMX was visualized and compared with the following 5 criteria: the Lipinski rule of 5 (molecular weight (MW) ≤ 500, HBD ≤ 5, HBA ≤ 10, LogP ≤ 5), the Veber filter (nrotb ≤ 10, TPSA ≤ 140 Å2), the Egan filter (LogP ≤ 5.88, TPSA ≤ 131.6 Å2), the Ghose rule (160 ≤ MW ≤ 480, -0.4 ≤ LogP ≤ 5.6, 40 ≤ MR ≤ 130, 20 ≤ no. atoms ≤ 70), and the Muegge filter (200 ≤ MW ≤ 600, −2 ≤ LogP ≤ 5, TPSA ≤ 150 Å2, no. rings ≤ 7, no. carbons > 4, no. heteroatoms > 1, nrotb ≤ 15, HBD ≤ 5, and HBA ≤ 10). The bioavailability score was calculated to predict the probability of 10% oral bioavailability or Caco-2 diffusion.

### 2.11. Statistical Analyses

Data were expressed as the mean ± standard deviation (SD) from 3 independent experiments. Statistical analyses were performed with GraphPad Prism 5 (GraphPad Software, San Diego, CA, USA). Statistical significance was presented as * *p* < 0.05, ** *p* < 0.01, and *** *p* < 0.001.

## 3. Results

### 3.1. Effect of CMX on the Proliferation of HFDPCs

**Figure 1:**
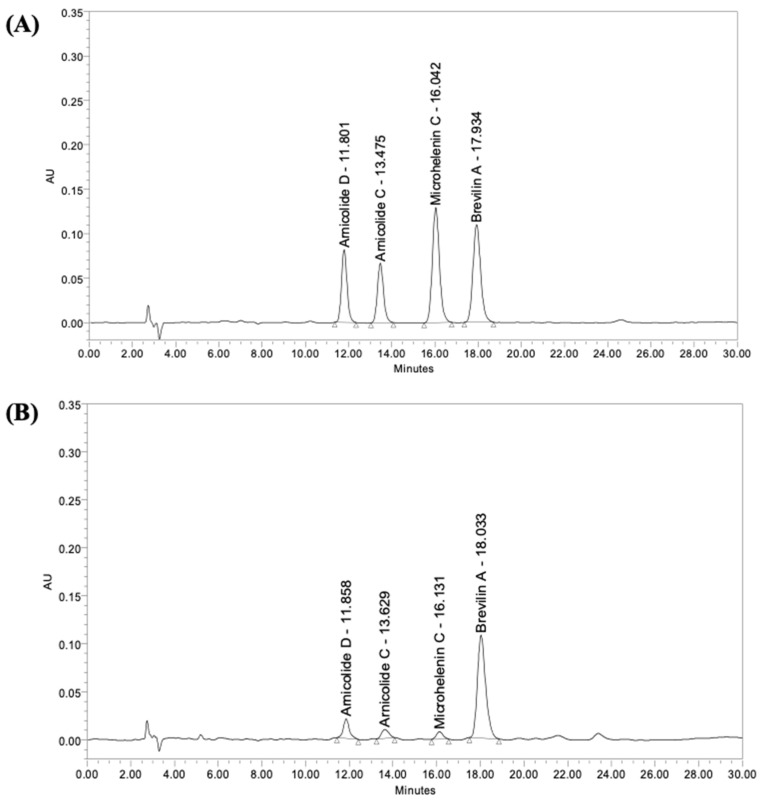
High-performance liquid chromatography (HPLC) chromatograms of (**A**) the standard of arnicolide D, arnicolide C, microhelenin C, and brevlin A, and (**B**) the emulsion extract of *Centipeda minima* (CMX). The column temperature was maintained at 40 °C and the wavelength was set at 224 nm.

**Figure 2:**
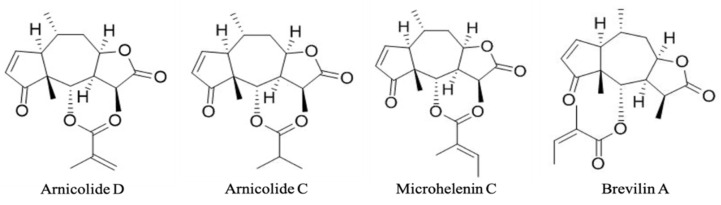
Chemical structures of arnicolide D, arnicolide C, microhelenin C, and brevilin A identified by HPLC-Q-TOF-MS.

The representative HPLC chromatograms of CMX are shown in Figure 1, and the four major compounds of CMX are marked with their retention time. These four compounds have been identified by HPLC-Q-TOF-MS as arnicolide D, arnicolide C, microhelenin C, and brevilin A, and their chemical structures are shown in Figure 2 [11]. The concentrations of these four major compounds were calculated by the calibration curve of the standard (1–100 µg/mL); the contents of each component in CMX are summarized in Table 2.

**Figure 3:**
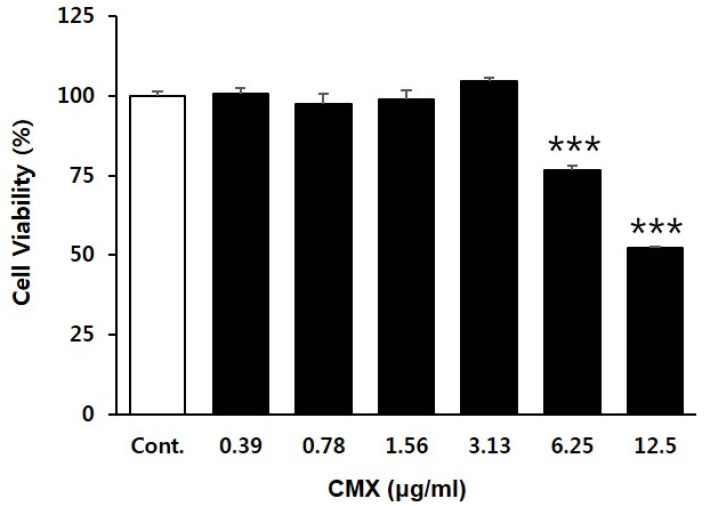
Effect of CMX on the viability of hair follicle dermal papilla cells (HFDPCs). Cells were treated with CMX at the indicated doses (0.39, 0.78, 1.56, 3.13, 6.25, and 12.5 µg/mL) for 24 h. Cell viability was analyzed using the WST-1 assay. Data are presented as the mean ± standard deviation (SD) from three independent experiments. *** *p* < 0.001.

We investigated the effect of CMX on the proliferation of HFDPCs. HFDPCs were treated with different concentrations (0, 0.39, 0.78, 1.56, 3.13, 6.25, and 12.5 µg/mL) of CMX for 24 h. Cell viability was estimated using the EZ-Cytox assay. The viability of HFDPCs treated with CMX is shown in Figure 3, and the percentages of cell viability were 100.5% (0.39 µg/mL), 97.5% (0.78 µg/mL), 98.9% (1.56 µg/mL), 104.4% (3.13 µg/mL), 76.5% (6.25 µg/mL), and 52.4%(12.5 µg/mL), respectively. Compared with that in the untreated HFDPCs, the CMX-treated cells (6.25 and 12.5 µg/mL) exhibited decreased viability. The highest increase in viability was observed at a concentration of 3.13 µg/mL CMX. Based on these data, 1.56 µg/mL and 3.13 µg/mL concentrations of CMX were chosen for further experiments.

### 3.2. CMX Increases the Expression of Genes Related to the WNT/β-Catenin Pathway in HFDPCs

**Figure 4:**
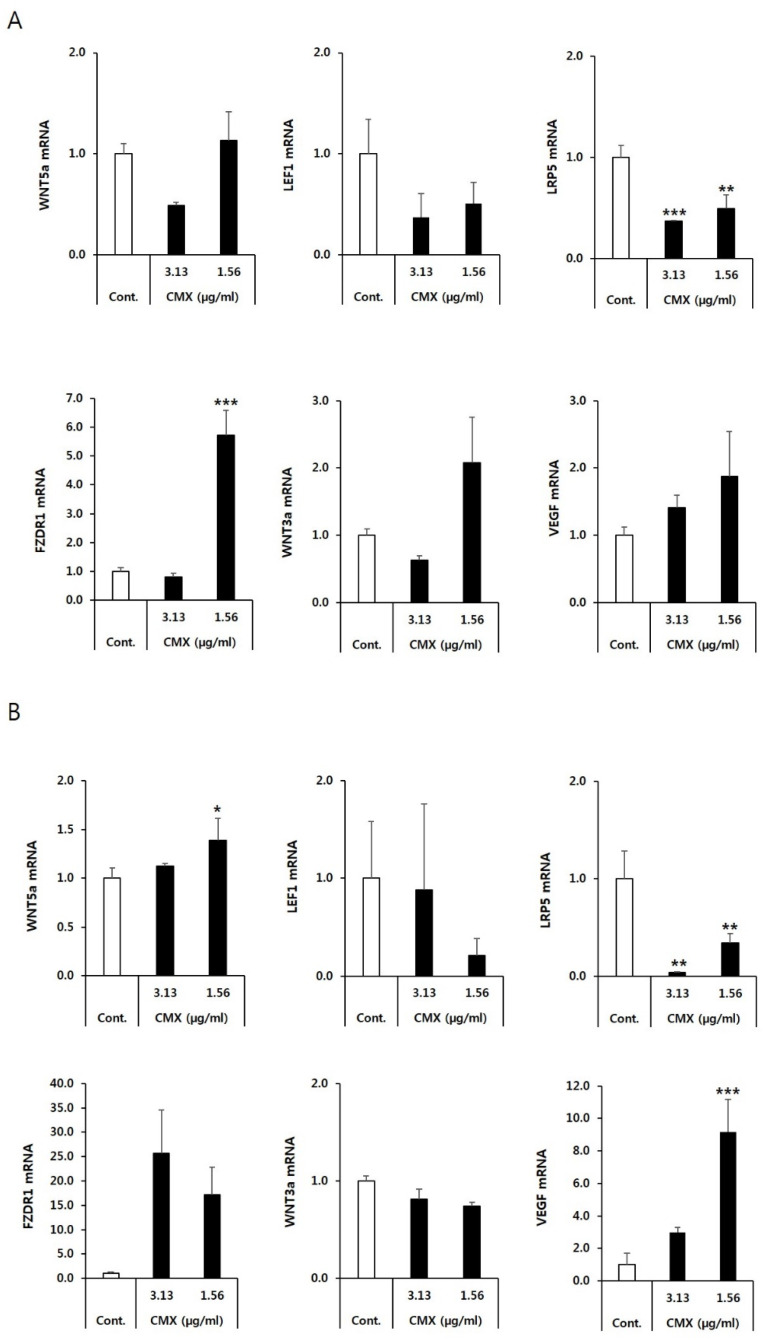
Expression of genes related to the Wnt signaling pathway and their signal transduction cascades in the hair follicles at 12 h (**A**) and 24 h (**B**). Hair follicle dermal papilla cells (HFDPCs) were seeded in six-well plates and treated with CMX (1.56 and 3.13 µg/mL). The transcript-level expression of hair growth-regulating factors was measured by quantitative real-time polymerase chain reaction (qPCR) using specific primers. GAPDH was used as an internal control. Data are presented as the mean ± standard deviation (SD) from three independent experiments. * *p* < 0.05, ** *p* < 0.01 and *** *p* < 0.001 vs. the control group.

The Wnt pathway plays an essential role in induction of hair follicle growth [17]. To determine whether CMX modulates the activity of the Wnt/β-catenin pathway in developing hair follicles, qRT-PCR was performed to examine the mRNA expression levels of Wnt/β-catenin signaling intermediaries, including Wnt ligands (Wnt3a and Wnt5a), Wnt receptors (FZDR1 and LRP5), Wnt transcription factors (LEF1), and VEGF, which is one of the most important mediators of the hair growth cycle. Comparative gene expression analysis was performed at 12 and 24 h of CMX treatment. A significant elevation in the expression of Wnt pathway factors started from 12 to 24 h. Compared with the untreated HFDPCs, cells treated with 1.56 µg/mL CMX exhibited the highest expression of Wnt5a and FZDR1 at 24 h, and the highest expression of Wnt3a at 12 h (Figure 4). These results suggest that CMX might possess the ability to stimulate hair follicles whose growth is regulated by Wnt/β-catenin-related genes. However, effect of CMX on LEF1 was not statistically significant.

### 3.3. CMX Enhances the Expression of Growth Factors Related to Hair Regeneration

**Figure 5:**
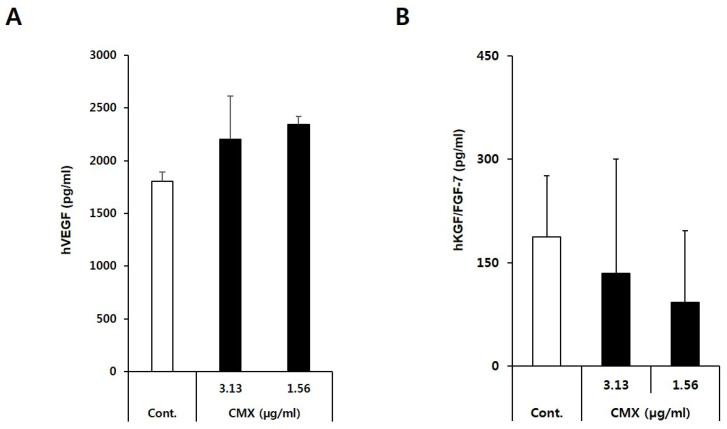
The secretion of growth factors in hair follicle dermal papilla cells (HFDPCs) treated with CMX. (**A**) Effect of CMX on hVEGF. (**B**) Effect of CMX on KGF/FGF-7. The concentrations of vascular endothelial growth factor (VEGF) and keratinocyte growth factor/fibroblast growth factor-7(KGF/FGF-7) secreted from HFDPCs in the CMX medium were analyzed by enzyme-linked immunosorbent assay (ELISA).

Hair regeneration is controlled by dermal papilla (DP) cells, which manage hair follicle cycling through secreted signaling factors [18]. Thus, we examined whether CMX could produce VEGF, a possible key angiogenic factor that mediates hair growth in HFDPCs. The amount of VEGF secreted from the the HFDPC growth medium was measured by ELISA, and the results are shown in Figure 5a. ELISA demonstrated that VEGF was released into the cell culture supernatant at higher levels in treated cells than in the untreated control. The VEGF concentrations of the CMX-treated group in HFDPCs were 2343.65 and 2202 pg/mL, which were 122% and 130% higher than those of the control group (100%), respectively. Furthermore, the growth factors secreted from the DP cells included basic fibroblast growth factor, insulin-like growth factor-1 (IGF-1), hepatocyte growth factor (HGF), fibroblast growth factor 1 (FGF1), and keratinocyte growth factor (KGF) [19]. Hair follicle growth was found to be inhibited by fibroblast growth factor (FGF) and transforming growth factor, while it was induced by insulin-like growth factor (IGF) and hepatocyte growth factor (HGF) [20]. Therefore, KGF/FGF secretion was monitored in the cell culture supernatants using ELISA. CMX-mediated KGF/FGF-7 secretions decreased to 71.8% (3.13 µg/mL) and 49% (1.56 µg/mL) compared with the control (100%) (Figure 5b). IGF-1 was not secreted from HFDPCs treated with CMX (data not shown). These results indicated that CMX enhanced the secretion of growth factors and that these proteins may stimulate hair follicle growth, thereby inducing hair regeneration.

### 3.4. CMX Activates Wnt/β-Catenin and ERK/JNK Signaling Pathways in HFDPCs

**Figure 6:**
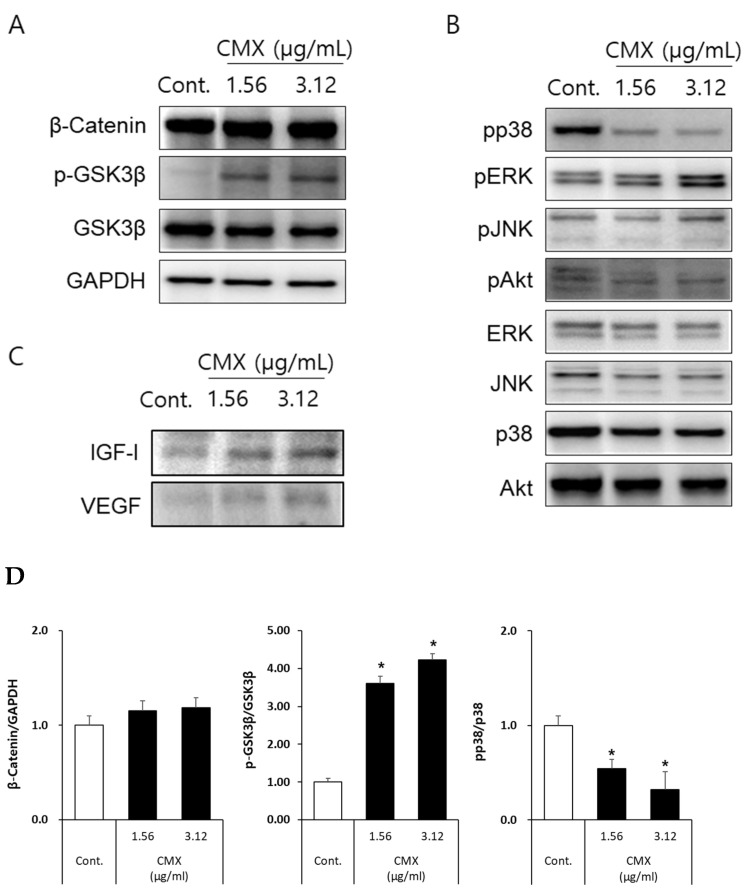

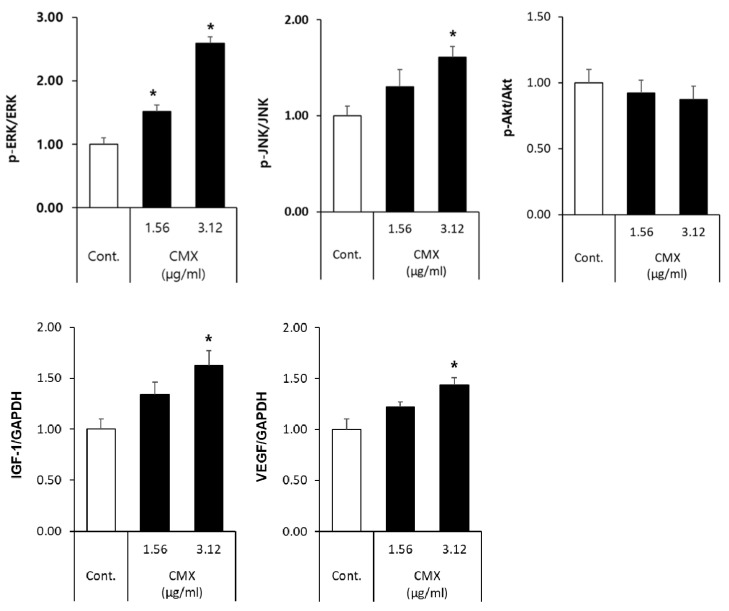
Effect of *Centipeda minima* extract (CMX) on the Wnt/β-catenin-related signal pathway in hair follicle dermal papilla cells (HFDPCs). (**A**) Effect of CMX on β-catenin, p-GSK3β and GSK3β. (**B**) Effect of CMX on the phosphorylation of MAPKs. (**C**) Effect of CMX on IGF and VEGF. (**D**) Quantified graphs for Western blots. HFDPCs were seeded into six-well plates (2 × 10^6^ cells/well) and treated with CMX at 1.56 and 3.13 µg/mL for 24 h. Whole-cell lysates were immunoblotted with the specific antibodies indicated on the left side of each panel. GAPDH served as an internal loading control. The bar chart displays the intensity of the immunoblot after normalization to the levels of GAPDH or the total form of phospho-protein. Data are presented as the mean ± standard deviation (SD) from three independent experiments. * *p* < 0.05 vs. the control group.

Wnt/β-catenin signaling plays a crucial role in the growth of DP cells [21,22]. The Wnt ligand is a secreted glycoprotein that binds to frizzled receptors, leading to the formation of a surface complex with LRP5/6. Activation of the Wnt receptor complex triggers the dissociation of the multifunctional kinase GSK-3β from the APC/Axin/GSK-3β-complex and, finally, β-catenin phosphorylation is inhibited. The unphosphorylated β-catenin accumulates in the cytosol, translocates into the nucleus, and binds to transcriptional factors, such as LEF-1/TCF1, which regulates the expression of Wnt/β-catenin signaling-regulated genes. Therefore, we investigated whether the Wnt/β-catenin signaling pathways were activated by CMX. First, the levels of β-catenin accumulation and phosphorylation status of GSK3β were analyzed. As shown in Figure 6a, immunoblotting revealed that CMX treatment increased β-catenin accumulation and the phosphorylation of GSK3β in a concentration-dependent manner in HFDPCs. Several studies have reported that phosphorylation of the ERK and PI3K/Akt pathways is implicated in DP cell proliferation [23,24]. Based on these results, we investigated the phosphorylation status of MAPKs (ERK, JNK, and p38) and Akt in HFDPCs. As shown in Figure 6b, the phosphorylation of ERK and JNK was increased in a concentration-dependent manner by CMX for 24 h, whereas, in the case of p38 phosphorylation, CMX significantly reduced the phosphorylation levels of stress-activated protein kinase (SAPK) in HFDPCs. Further, we analyzed the phosphorylation status of Akt; the phosphorylation of Akt was not affected by CMX treatment for 24 h in HFDPCs. Additionally, DP cells are known to secrete growth factors for the regulation of hair growth via autocrine and paracrine factors [23,24,25]. Therefore, we analyzed whether IGF and VEGF levels were increased by CMX treatment. As shown in Figure 6c, the expression of IGF and VEGF proteins was upregulated by CMX in a concentration-dependent manner for 24 h. Collectively, these results indicated that CMX significantly increased the secretion of growth factors such as IGF and VEGF, which are regulated by Wnt/β-catenin and the activation of ERK and JNK signaling pathways in HFDPCs.

### 3.5. CMX Increases β-Catenin Localization in HFDPCs

**Figure 7:**
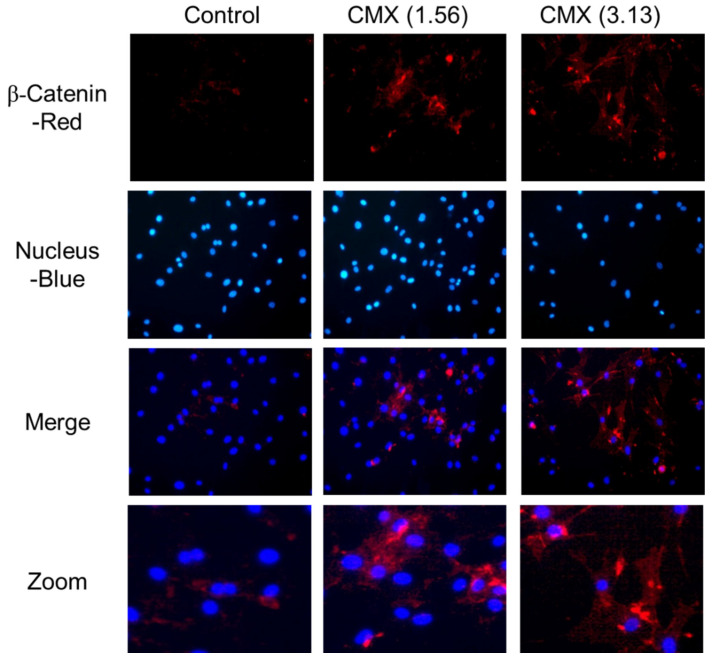
Effect of *Centipeda minima* extract (CMX) on the accumulation of β-catenin in hair follicle dermal papilla cells (HFDPCs). HFDPCs were immunocytochemically stained with β-catenin antibody (red color, first row), and the corresponding images of DAPI nuclear staining are shown (blue color, second row); merged images are shown in the bottom panel. Original magnification: 20×.

When cells are not stimulated by Wnt, cytoplasmic β-catenin is phosphorylated and degraded by kinases called casein kinase-I and GSK3 in the Axin and APC protein complexes. However, when Wnt is activated by external stimulation, the degradation of β-catenin is inhibited; therefore, the intracellular β-catenin level is increased and stabilized, and β-catenin translocates into the nucleus. Therefore, we performed an immunofluorescence assay to examine the accumulation and localization of β-catenin in HFDPCs after treatment with CMX for 24 h. CMX treatment resulted in a significant accumulation of β-catenin in the nucleus in a concentration-dependent manner compared with the DMSO control. This result correlated with the increase in β-catenin protein levels revealed by immunoblotting (Figure 7).

### 3.6. Pharmacokinetic and Drug-Likeness Profiles of CMX

**Figure 8:**
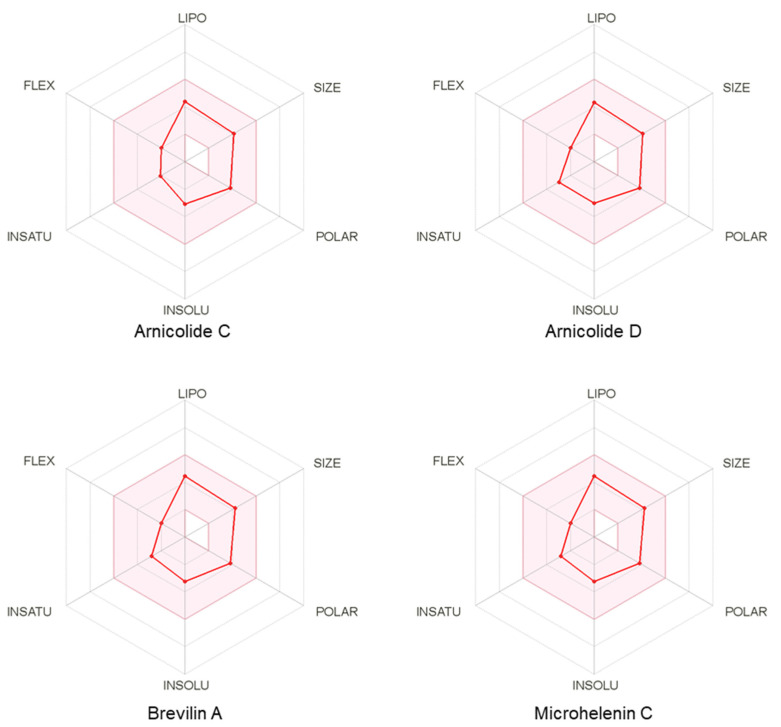
Bioavailability radar of *Centipeda minima* (CMX) compounds. The pink area represents the optimal range for each property (LIPO, size, polarity, solubility, saturation, and flexibility.

Computational predictions of drug-likeness together with ADME property predictions can assess the possibility of potential lead compounds [26]. We evaluated the pharmacokinetic and drug-likeness properties of CMX using the web-based tool SwissADME. The results showed that all compounds were estimated to have high gastrointestinal absorption, which can be considered a favorable advantage in the case of oral administration (Table 3). We found that none of the compounds of CMX interacted with the cytochrome P450 (CYP) enzyme, indicating that these compounds did not affect the biotransformation process. Drug-likeness is a qualitative inspection of a compound’s physicochemical or structural properties to investigate the likelihood of the compound as an oral drug-like candidate [27]. The result of the drug-likeness assessment showed that all compounds in CMX followed all the prominent drug-like rules, including Lipinski’s rule of five, the Ghose rule, the Veber filter, the Egan filter, and the Muegge filter, indicating that these compounds had desirable properties similar to those of orally administered drugs. The polygon of physicochemical space also showed that all parameters fell within the optimal range, and therefore, all the compounds possessed good oral bioavailability (Figure 8). The satisfactory bioavailability score of CMX compounds suggests that it is expected to exhibit high gastrointestinal absorption.

## 4. Discussion

Hair is one of the most unique features of mammals and is an indicator of individual health, as it serves multiple physiological functions, including protecting the body from environmental damage and maintaining body temperature [28]. The hair follicle, a specific appendage of the skin, is composed of epidermal (epithelial) and dermal (mesenchymal) compartments, and their reciprocal interaction plays a crucial role in the growth of the hair follicle development and the hair cycle [29]. HF is a perfect mammalian model system for understanding the intra- and inter-cell signaling pathways that may be investigated in other tissues.

*C. minima* is a well-known medicinal plant used for the treatment of whooping cough, cold, nasal allergy, asthma, diarrhea, and malaria in Chinese medicine. It is widely found in China, Korea, and Southeast Asia and is distributed over certain areas in Australia and India. Twelve major chemical constituents such as flavonoids, polyphenolic acid, and sesquiterpene lactones have been identified [8,9]. Brevilin A corresponds to the structure of sesquiterpene lactones, and it is a major constituent of *C. minima* [9]. CMX was prepared by extracting sesquiterpene lactones such as brevilin A with high purity and high content. Recently, CMX, an emulsion extract of *C. minima*, was shown to induce hair regeneration, and increased hair count has been demonstrated by a previous study [11]. The patients in the CMX group showed significant improvements in the total, terminal, and anagen hair counts. Therefore, it is important to understand the molecular mechanisms and effects of CMX in HFDPCs. DPs are specialized fibroblasts that secrete several molecules, such as wingless-int (Wnt), sonic hedgehog (Shh), and bone morphogenetic protein (BMP), which signal to epithelial cells [30,31,32]. Canonical Wnt signaling plays an essential role during the anagen-promoting process [22]. Therefore, factors affecting DPC function in HFDPCs are important in potential hair loss therapies [33,34].

Wnt/β-catenin signaling, among other signaling systems, has been implicated in the regulation of hair follicle morphogenesis and regeneration [7,22,35]. Various Wnt (wingless type) ligands are involved in hair cycling. Wnt-responding stem cells in the bulge produce Wnt1, Wnt4, and Wnt7b during the telogen phase. Wnt6, Wnt10a, and Wnt10b are strongly expressed in the dermal papilla during the telogen to anagen transition. During anagen, other Wnts, including Wnt5a and Wnt5b, are mainly expressed in the peripheral layers of the dermal papilla [36]. The secreted Wnt protein is a ligand that binds to the frizzled family receptor, which then passes the biological signal to the intracellular protein “disheveled” (Dsh) inside the cell. Disheveled protein causes the accumulation of β-catenin in the cytoplasm and its eventual translocation into the nucleus acts as a transcriptional co-activator for the T-cell factor (TCF)/lymphoid enhancer factor (LEF) transcription factors, a phenomenon that results in the induction of Wnt-regulated genes [7]. Therefore, we examined the transcript-level expression of the intermediaries of the Wnt cascade at 12 and 24 h. We found that the transcript-level expression of Wnt5a, VEGF, and FZDR was increased at 12 and 24 h. The expression of Wnt3a was upregulated at 12 h but not at 24 h. The expression of Wnts 10a, 10b, and 5a was specifically upregulated in hair follicles during the early morphogenetic phase [30]. Wnt3a is an inductive signal that maintains HFDPCs in the anagen phase [22]. Wnt5a is a key target gene of Shh in hair follicle morphogenesis, although Shh is not required for the positioning of follicles [36]. Millar et al. suggested that the observed decrease in the hair length of Wnt3 and Dvl2 transgenic animals could be due to a premature transition of follicles from the anagen to the catagen phase of the hair growth cycle or could be caused by defects in the proliferation or differentiation of hair matrix cells or cells derived from them [31].

We observed that the levels of β-catenin and GSK3β, which are key intermediaries of the Wnt/β-catenin signaling pathway, were increased. Additionally, the MAPK and Akt signaling pathways are major regulators of cell proliferation. Our results showed that ERK and JNK were phosphorylated in CMX-treated cells in a dose-dependent manner. We demonstrated that these proteins are involved in the proliferation of HFDPCs. Follicle DP cells, located at the base of hair follicles, regulate the hair cycle by responding externally to stimuli and signals conveyed through cytokines and junctions [33].

HDPFCs relay signals to epithelial cells through secreted molecules such as Wnt, Shh, and BMP during the hair cycle [34]. Moreover, the cells show the active expression of secreted molecules, such as growth factors, that regulate the proliferation of neighboring epidermal cells and differentiation via epithelial–mesenchymal interactions [8]. Hair follicles require sufficient nutrients for hair growth during the anagen phase. VEGF, which induces angiogenesis, is important for supplying nutrients to the hair follicles [36]. Our studies on growth factors have shown that VEGF is secreted in a dose-dependent manner in CMX-treated cells (RNA and protein levels). The level of IGF protein was also increased by CMX in HFDPCs.

**Figure 9:**
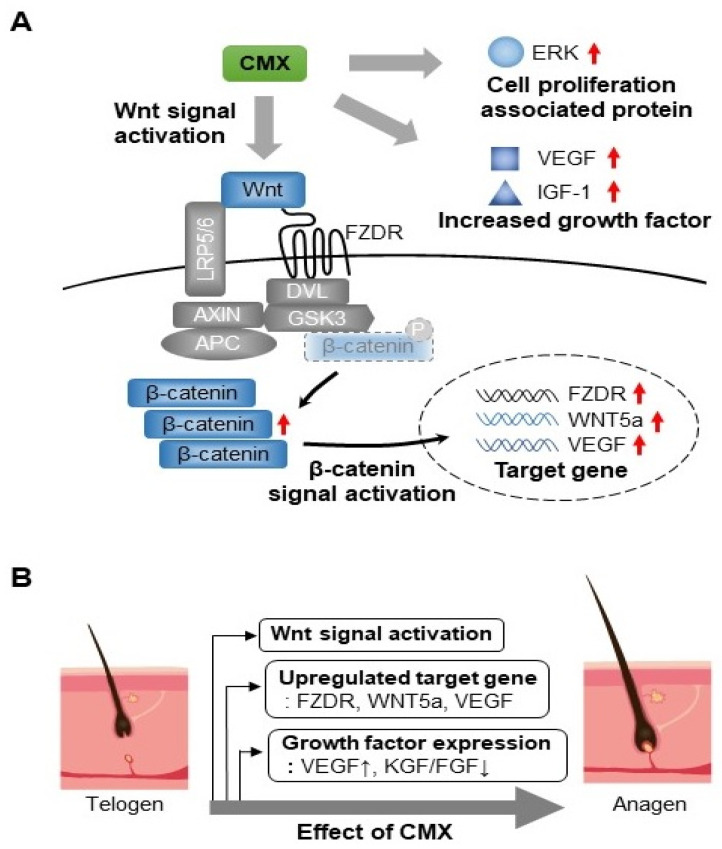
The effect of *Centipeda minima* extract (CMX) on hair growth. (**A**) Wnt activation and related signal pathways. The above figure shows CMX as a major effector of the Wnt/β-catenin signaling pathway. In the absence of the Wnt ligand, the levels of β-catenin are low in the cytoplasm. However, when the Wnt ligand binds to frizzled (FZDR), LRP5/6 (low-density lipoprotein receptor-related protein 5 or 6), and Axin disruption complexes, β-catenin accumulates in the cytoplasm, migrates into the nucleus, interacts with the TCF transcription factor, and activates the target genes, such as Wnt5a/FZDR/VEGF. Additionally, CMX enhances ERK activation and the expression of growth factors, such as VEGF and IGF-1, in the extracellular medium. (**B**) Effect of CMX on anagen stimulation. The activation of the Wnt/β-catenin signaling pathway promotes anagen induction.

β-catenin is an important regulator of Wnt-induced gene transcription. Using immunocytochemistry, we confirmed that β-catenin was significantly localized in the nucleus when the Wnt pathway was activated by CMX. Our results indicated that CMX upregulated the expression of growth factors such as VEGF and IGF via the Wnt/β-catenin, ERK, and JNK signaling pathways in HFDPCs. Taken together, our findings demonstrated that CMX can induce hair proliferation via the Wnt/β-catenin, ERK, and JNK signaling pathways, and secreted growth factors (Figure 9). In addition, when the content of brevilin A was calculated by considering the concentration of CMX in treated cells (3.13 and 1.56 µg/mL), it corresponded to 0.62 and 1.25 ng/mL, respectively, and it was considered to have an effect at a very low concentration. Additionally, the bioavailability of CMX compounds resulted in high gastrointestinal absorption. Therefore, CMX could be used as a new therapeutic agent to facilitate hair growth.

## 5. Conclusions

This study showed that CMX regulates signaling in HFDPCs and stimulates hair regeneration through the Wnt/β-catenin, ERK, and JNK signaling pathways, resulting in the upregulation of VEGF and IGF factors. In conclusion, our study suggests the use of CMX, which has little toxicity, as an effective treatment for alopecia and highlights the possibility of cosmeceuticals in promoting hair regeneration.

## Figures and Tables

**Table 1:** List of primers for real-time polymerase chain reaction.

Target	Forward Primer	Reverse Primer
WNT5a	TCCACCTTCCTCTTCACACTGA	CGTGGCCAGCATCACATC
LEF1	CAGTGACGAGCACTTTTCTC	CGTGATGGGATATACAGGCT
LRP5	GCTGTACCCGCCGATCCT	GGCGCCATTCCTCGAAT
FZDR1	CCAAGAGAGGAGCCGAGA	CGGCACAAAGTTCCCAG
WNT3a	CCTCAAGGACAAGTACGACA	GGCACCTTGAAGTAGGTGTA
VEGF	ATGACGAGGGCCTGGAGTGTA	CCTATGTGCTGGCCTTGGGA
GAPDH	GGTGGTCTCCTCTGACTTCAACA	GTTGCTGTAGCCAAATTCGTTGT

Wnt5a: wingless type MMTV integration site family, member 5A, Lef1: lymphoid enhancer-binding factor-1, LRP5: low-density lipoprotein receptor-related protein 5, FZDR1: frizzled receptor 1, Wnt3a: wingless type MMTV integration site family, member 3, VEGF: vascular endothelial growth factor, GAPDH: glyceraldehyde 3-phosphate dehydrogenase.

**Table 2:** Contents of arnicolide D, arnicolide C, microhelenin C, and brevilin A in CMX.

	Arnicolide D	Arnicolide C	Microhelenin C	Brevilin A
Contents (µg/g)	147.5	39.8	45.7	400.5

**Table 3:** Pharmacokinetics and drug-likeness properties of *Centipeda minima* extract (CMX).

	Arnicolide C	Arnicolide D	Brevilin A	Microhelenin C
**Pharmacokinetics**				
GI absorption	High	High	High	High
CYP1A2 inhibitor	No	No	No	No
CYP2C19 inhibitor	No	No	No	No
CYP2C9 inhibitor	No	No	No	No
CYP2D6 inhibitor	No	No	No	No
CYP3A4 inhibitor	No	No	No	No
Log Kp (cm/s)	−6.81	−6.86	−6.78	−6.78
**Drug-likeness**				
Lipinski	0	0	0	0
Ghose	0	0	0	0
Veber	0	0	0	0
Egan	0	0	0	0
Muegge	0	0	0	0
Bioavailability score	0.55	0.55	0.55	0.55

## Data Availability

Not applicable.
